# *Olea europaea* L. Leaves as a Source of Anti-Glycation Compounds

**DOI:** 10.3390/molecules29184368

**Published:** 2024-09-14

**Authors:** Marzia Vasarri, Maria Camilla Bergonzi, Emilija Ivanova Stojcheva, Anna Rita Bilia, Donatella Degl’Innocenti

**Affiliations:** 1 Department of Chemistry, University of Florence, Via Ugo Schiff 6, 50139 Sesto Fiorentino, Italy; marzia.vasarri@unifi.it (M.V.); annarita.bilia@unifi.it (A.R.B.); 2 Department of Experimental and Clinical Biomedical Sciences, University of Florence, Viale Morgagni 50, 50134 Florence, Italy; donatella.deglinnocenti@unifi.it; 3 Natac Biotech SL, Rita Levi Montalcini 14, 28906 Getafe, Spain; eivanova@natacgroup.com

**Keywords:** *Olea europaea* L. leaves extracts, oleuropein, triterpenes, albumin glycation, gelatin glycation, anti-glycation, advanced glycation end products

## Abstract

High concentrations of advanced glycation end products (AGEs) have been linked to diseases, including diabetic complications. The pathophysiological effects of AGEs are mainly due to oxidative stress and inflammatory processes. Among the proteins most affected by glycation are albumin, the most abundant circulating protein, and collagen, which has a long biological half-life and is abundant in the extracellular matrix. The potential cellular damage caused by AGEs underscores the importance of identifying and developing natural AGE inhibitors. Indeed, despite initial promise, many synthetic inhibitors have been withdrawn from clinical trials due to issues such as cytotoxicity and poor pharmacokinetics. In contrast, natural products have shown significant potential in inhibiting AGE formation. *Olea europaea* L. leaves, rich in bioactive compounds like oleuropein and triterpenoids, have attracted scientific interest, emphasizing the potential of olive leaf extracts in health applications. This study investigates the anti-glycation properties of two polyphenol-rich extracts (OPA40 and OPA70) and a triterpene-enriched extract (TTP70) from olive leaves. Using in vitro protein glycation methods with bovine serum albumin (BSA)–glucose and gelatin–glucose systems, this study assesses AGE formation inhibition by these extracts through native polyacrylamide gel electrophoresis (N-PAGE) and autofluorescence detection. OPA40 and OPA70 exhibited strong, dose-dependent anti-glycation effects. These effects were corroborated by electrophoresis and further supported by similar results in a gelatin–glucose system. Additionally, TTP70 showed moderate anti-glycation activity, with a synergistic effect of its components. The results support the real possibility of using olive leaf bioproducts in ameliorating diabetic complications, contributing to sustainable bio-economy practices.

## 1. Introduction

Advanced glycation end products (AGEs) are formed through the non-enzymatic Maillard reaction of reducing sugars with amino acids in proteins, lipids, or DNA and they are able to form covalent cross-links with proteins [1,2]. The glycation process is complex and slow, but the presence and accumulation of AGEs in many cell types affect the extracellular and intracellular structure and function and cause spontaneous damage to proteins in physiological systems [3,4]. Although AGEs are produced physiologically in the human body, when present in high concentrations, they can cause a number of pathological states, including diabetic complications [5,6]. For this reason, they are sometimes referred to as glycotoxins because they can be toxic to the body if present for a prolonged period [7]. Furthermore, AGEs are not only produced endogenously, but they can also be ingested through food. Modern dietary trends have led to an increase in AGE consumption associated with an increase in metabolic dysfunction, obesity, and diabetes, which in turn facilitates the production of endogenous AGEs in the body [8]. In general, the pathophysiological effects of AGEs may be related to several mechanisms of action, including the oxidative stress that may lead to the damage of various cellular components and the activation of the inflammatory process [4,9,10].

Among the proteins most easily affected by the glycation process are albumin and collagen. In fact, albumin is the most abundant circulating protein in the blood, which is why it is constantly exposed to numerous metabolites and conditions, e.g., hyperglycemia, that can facilitate glycation [11]. Moreover, collagen, being abundantly present in the extracellular matrix (ECM) and possessing a long biological half-life, is also easily accessible to glycation with significant biological late consequences in diabetes complications and aging [12].

The crucial role of AGEs in the onset of many disease conditions has initiated the process of identifying and developing AGE inhibitors that suppress their formation [13,14]. Today, there is growing interest in agents with anti-glycation activity that could play a key role in preventing and ameliorating AGE-mediated health problems. Currently known AGE inhibitors can generally be divided into two groups: synthetic compounds and natural products. Aminoguanidine is the first drug synthesized with excellent anti-glycation properties, but not approved for clinical use due to its cytotoxicity. The pharmacokinetics, safety, and efficacy of many other synthetic compounds, despite their inhibitory ability to form AGEs, have not been satisfactory, resulting in their withdrawal from clinical trials [15].

Over the past 30 years, there has been a significant increase in anti-glycation agents of natural origin. So far, certain plant extracts and their bioactive compounds have been evaluated for their activity against the formation of AGEs. In view of this, natural products possessing strong inhibitory properties on AGE formation are a source of inspiration for further research of inhibitor AGE formation and preventive drugs against AGE-related disorders and diseases [16].

*Olea europaea* L. is one of the most valuable fruit trees producing olive oil, which is important for human nutrition. Several bioactive secondary metabolites belonging to different chemical classes and with various nutritional and pharmacological properties have been identified in the bark, root, wood, and leaf of this plant [17]. Among the main components of olive plants are phenolic compounds, of which oleuropein (Ole), demethyl-Oleuropein, ligstroside, and oleoside represent the predominant phenolic oleosides. Ole is widely described in the literature for its innumerable beneficial properties for human health, mainly associated with its antioxidant and radical scavenger capacity [18,19].

Numerous benefits for human health have also been attributed to the presence of sterols and triterpenes (TTPs) in olive leaves [20,21,22,23,24]. The TTP content is higher in the leaves than in the fruit and depends on the variety. The leaf contains high amounts of oleanolic acid (3.0–3.5% dry weight), a significant concentration of maslinic acid (0.50–0.75% dry weight), and lower levels of ursolic acid (0.20–0.25% dry weight), erythrodiol, and uvaol, which are present in comparable amounts in the range of 0.05 to 0.15 per cent dry weight [25].

Moreover, olive tree leaves have been used since ancient times in traditional medicine as a natural remedy. Overall, olive tree leaves have attracted increasing attention from the scientific community as a source of interesting bioactive compounds potentially exploitable for human health [23,26,27,28,29,30]. Since Ole and oleanolic acid are the primary constituents of olive products, olives are used as a natural cure and olive oil is a practical component of the Mediterranean diet.

Furthermore, the idea of using and promoting the recovery of generally wasted products, such as leaves in olive oil production, is an innovative intervention in the circular and sustainable bio-economy. Indeed, it is necessary to consider the growing demand for natural compounds that can be used for human health and well-being while respecting the environment. The extraction of the fraction rich in bioactive metabolites from waste material is important in terms of environmental impact. Olive leaves are produced from the olive oil industry in high quantity, and they have to be removed from the fields and mills. However, olive leaves represent a precious resource to be transformed into by-products in the process of valorizing these biomasses [31]. 

In light of these considerations, in this work, the anti-glycation role of some extracts from *Olea europaea* L. leaves was investigated. In particular, the anti-glycation effects of two extracts with a high content of total polyphenols (OPA-EXTs), one containing 40% *w*/*w* (OPA40) and one containing 70% *w*/*w* (OPA70), were compared. Furthermore, the anti-glycation effect of *Olea europaea* L. leaf extract enriched in pentacyclic triterpenes (TTP70) was compared with the anti-glycation effect of its main active component, the pentacyclic triterpenoid oleanolic acid (OA).

The effect of the extracts against AGE formation was evaluated by an in vitro protein glycation method, using either the bovine serum albumin (BSA)–glucose system or the gelatin–glucose system, as both proteins are readily involved in the glycation process in vivo. AGE formation in the presence/absence of olive leaf extracts was assessed by both native polyacrylamide gel electrophoresis (N-PAGE) and autofluorescence detection.

## 2. Results and Discussion

### 2.1. Characterization of OPA-EXTs

OPA40 and OPA70 are Ole-enriched fractions containing 41.67 ± 0.55 g and 66.59 ± 1.27 g per 100 g of extract, respectively. OPA40 and OPA70 contain a wide variety of phenolic compounds, which account for 49.63 ± 0.69% and 75.32 ± 1.58% *w*/*w*, respectively. The complete characterization of the polyphenols is provided in Table 1. The chromatographic profile of OPA40 is reported in Figure S1 of the Supplementary Material.

### 2.2. Characterization of TTP70 Extract

*Olea europaea* L. leaves have been reported to be a rich source of both free and fatty acid esterified pentacyclic triterpenic acids and triterpenols. Of these, oleanolic, ursolic, and maslinic acids are the most important triterpene acids in olive leaves, just as uvaol and erythrodiol are triterpene alcohols [24].

In this work, the TTP70 extract contains mostly these compounds in accordance with the literature.

Specifically, in the TTP70 extract, maslinic acid, oleanolic acid, ursolic acid, uvaol, and erythrodiol were identified (Table 2); the percentage of TTPs was 65.34 ± 1.06% *w*/*w*. The chromatographic profile of TTP70 is reported in Figure S2 of the Supplementary Material.

### 2.3. The Anti-Glycation Role of Olea europea L. Leaf Extracts 

Despite significant developments in antidiabetic therapy, most antidiabetic drugs available on the market today act mainly to control blood glucose levels. Despite this, diabetic individuals have a higher risk of developing complications often related to AGE formation. It is therefore important for scientific research to identify agents capable of counteracting protein AGE formation [1].

In this study, the *Olea europaea* L. leaf extracts were investigated as possible agents against albumin and gelatin glycation, as two accessible substrates to protein glycation during prolonged disease states such as hyperglycemia. 

#### 2.3.1. The Inhibitory Effect of OPA-EXTs in the In Vitro Albumin–Glycation System (A-AGE)

As the most abundant circulating protein in the blood, albumin is easily exposed to various conditions, such as hyperglycemia, that may facilitate its glycation.

**Figure 1:**
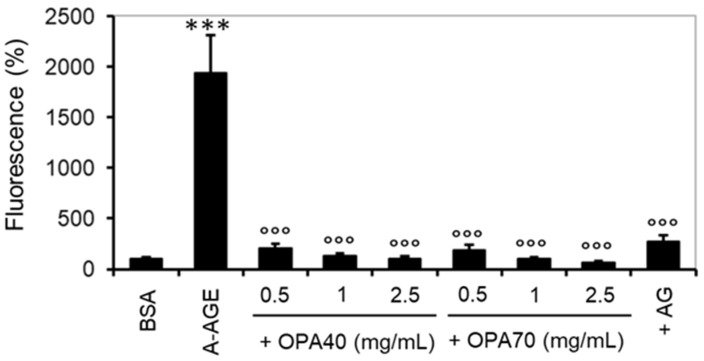
In vitro glycation of albumin and the inhibitory effect of OPA40 and OPA70 on A-AGE formation. Relative fluorescence intensity (λex/λem 335/385 nm) of unglycated albumin (BSA), A-AGE, and A-AGE with OPA40 (0.5–2.5 mg/mL), OPA70 (0.5–2.5 mg/mL), or AG (10 mM) obtained at 60 °C for 72 h. All values are reported as percentage ratio with respect to unglycated BSA. Data are the mean of three independent experiments. Error bars represent standard deviation. Tukey’s test: *** *p* < 0.001 vs. unglycated BSA; °°° *p* < 0.001 vs. A-AGE formed in the absence of the extracts.

Albumin glycation was performed in vitro by incubating BSA (1 mg/mL) with Glc (500 mM) for 72 h at 60 °C, according to the experimental model described previously [32,33]. The formation of A-AGE was verified by measuring the intrinsic fluorescence of the glycation products at a 335 and 385 nm excitation and emission wavelength. As shown in Figure 1, the fluorescence of A-AGE is approximately 20 times (1939 ± 371%) higher than that of unglycated albumin (BSA). BSA incubated in the absence of Glc for 72 h at 60 °C was used as a reference sample. 

To evaluate the effect of OPA40 and OPA70 extracts on albumin glycation, the formation of A-AGE was examined by incubating at 60 °C for 72 h BSA (1 mg/mL) and Glc (500 mM) in the presence of increasing doses of OPA40 and OPA70 (from 0.5 to 2.5 mg/mL) and by monitoring the formation of fluorescent products. Both OPA-EXTs inhibited A-AGE formation in a dose-dependent manner, as represented in Figure 1. Specifically, OPA40 inhibited A-AGE formation by approximately 9-fold (209 ± 39%) and 14-fold (137 ± 21%) at the concentration of 0.5 and 1 mg/mL, respectively (corresponding to 0.2 mg/mL and 0.4 mg/mL Ole, respectively). Comparably, OPA70 reduced A-AGE formation by 10-fold (185 ± 52%) and 18-fold (104 ± 15%) at the concentration of 0.5 and 1 mg/mL, respectively (corresponding to 0.35 mg/mL and 0.7 mg/mL of Ole). At the highest tested OPA-EXT concentration of 2.5 mg/mL (corresponding to 1 mg/mL and 1.75 mg/mL Ole in the OPA40 and OPA70, respectively), the formation of A-AGE was reduced approximately 19-fold (102 ± 20%) and 28-fold (67 ± 12%), respectively. Note that both OPA-EXTs showed no autofluorescence at the wavelengths used for A-AGE detection.

In addition, aminoguanidine (AG), a potent synthetic inhibitor of protein glycation, was used in this study as a positive control for the inhibition of A-AGE formation. As shown in Figure 1, AG (10 mM) reduced A-AGE formation by approximately 7-fold (274 ± 62%). It is noteworthy that both effects exhibited a greater anti-glycant role than the positive control. This makes both extracts promising anti-glycating agents with potential for exploitation in human health.

**Figure 2:**
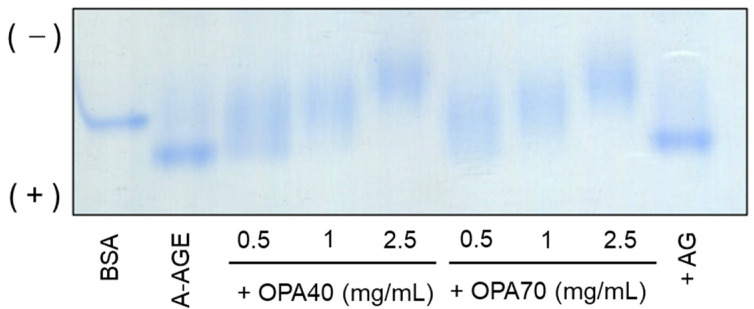
Representative N-PAGE image of unglycated albumin (BSA) and A-AGE obtained at 60 °C for 72 h in the absence or presence of OPA40 and OPA70 extracts. A-AGE obtained in the presence of AG was used as a protein glycation inhibition control. The symbols (+) and (−) represent the anode and cathode in the N-PAGE gel, respectively.

During the process of protein glycation, the presence of glucose causes a conformational change in albumin, resulting in early glycation products. The isoelectric point of albumin is 4.9, but during this process, the condensation of the positive residues (arginine and lysine) with glucose reduces the cationic charges of the glycation products, compromising the isoelectric point of albumin [11]. Since the loss of positive charges favors the electrophoretic migration from the cathode to the anode at pH 8.3, the formation of A-AGE can also be proved by the N-PAGE technique. As depicted in Figure 2, the A-AGE electrophoretic migration consistently increased with respect to unglycated albumin (BSA) as expected by the condensation of positive residues. This result is consistent with the autofluorescence results just discussed. As can be seen from the electrophoresis image, the highest concentration of the extract used, 2.5 mg/mL, results in significantly reduced migration compared to non-glycated BSA. This phenomenon could be due to the high amount (2.5 mg/mL) of extract molecules applied to the electrophoretic run. The electrophoretic analysis depends on the purity of the sample; the protein sample could have molecules that interfere with the solubility and electrophoretic mobility of the protein (1 mg/mL). N-PAGE is a technique that involves protein migration under non-denaturing conditions, so the proteins in the applied sample are not subjected to denaturation and possible hydrophobic aggregations or interactions that may alter sample migration [34].

Overall, these results demonstrate that OPA-EXTs inhibited AGE formation in the albumin–glycation system, suggesting a potent anti-glycation property comparable to that of the well-known synthetic AG inhibitor. Therefore, these data support the real possibility of a complementary use of natural molecules in the treatment of diabetes complications. The need for anti-glycation agents that are effective and known to be already used in traditional medicine [35], and thus free of toxicity, highlights this study and the potential use of OPA-EXTs against diabetic complications. 

#### 2.3.2. The Inhibitory Effect of OPA-EXTs in the In Vitro Gelatin–Glycation System (G-AGE)

Since collagen is one of the most abundant proteins in the extracellular matrix and possesses a long biological half-life, it is easily accessible to glycation with significant biological late consequences in diabetes complications and aging [36]. Therefore, in this work, the possible effect of OPA-EXTs on protein glycation was investigated using the in vitro gelatin–glucose system. 

Specifically, gelatin glycation was performed in vitro by incubating gelatin (1 mg/mL) with Glc (500 mM) from 0 to 96 h at 45 °C. Indeed, gelatin is resistant to thermal denaturation and compatible with the used temperature that can accelerate the rate of glycation [37]. 

To verify the G-AGE formation, the intrinsic fluorescence intensity of the glycation products was detected at a 335 and 385 nm excitation and emission wavelength. 

**Figure 3:**
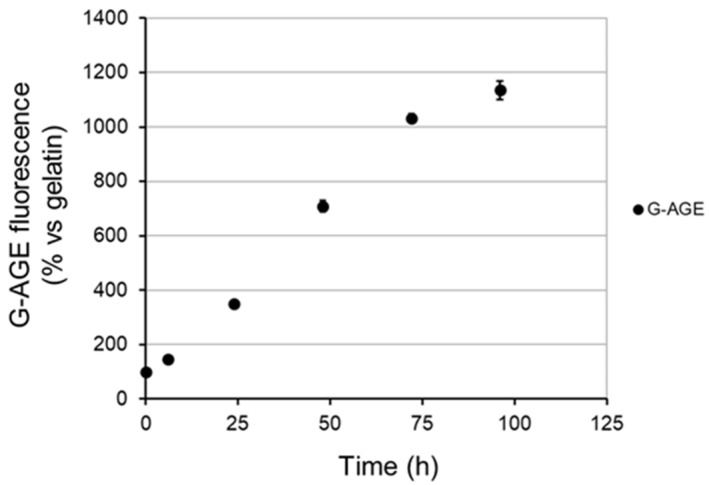
Time-dependent formation of G-AGE. The relative fluorescence intensity of G-AGE was measured from time 0 to 96 h at 335 and 385 nm excitation and emission wavelength. Values are reported as percentages compared to the fluorescence intensity of gelatin at time 0. Values are the mean ± standard deviation of three experiments.

As shown in Figure 3, the G-AGE formation depended on the incubation time. At 24 h and 48 h of incubation, the fluorescence intensity was approximately 3.5-fold (350 ± 5%) and 7-fold (708 ± 21%) higher, respectively, than that of gelatin at the starting time (t0). After 72 h, the fluorescence intensity of G-AGE was 10-fold (1032 ± 15%) higher than that of gelatin at t0, while at 96 h, the fluorescence peak was mostly unchanged from 72 h.

Given the time dependence of the protein glycation process, it was decided to use 72 h as a significant time point for the formation of G-AGE to evaluate the effect of the two OPA-EXTs on gelatin glycation. 

Specifically, the formation of G-AGE was examined by incubating at 45 °C for 72 h gelatin (1 mg/mL) and Glc (500 mM) in the presence of increasing doses of OPA40 and OPA70 (from 0.5 to 2.5 mg/mL) and by monitoring the formation of fluorescent products at a 335 and 385 nm excitation and emission wavelength. Gelatin incubated in the absence of Glc for 72 h at 45 °C was used as a reference sample.

Note that OPA-EXTs showed no autofluorescence at the wavelengths used for G-AGE detection.

**Figure 4:**
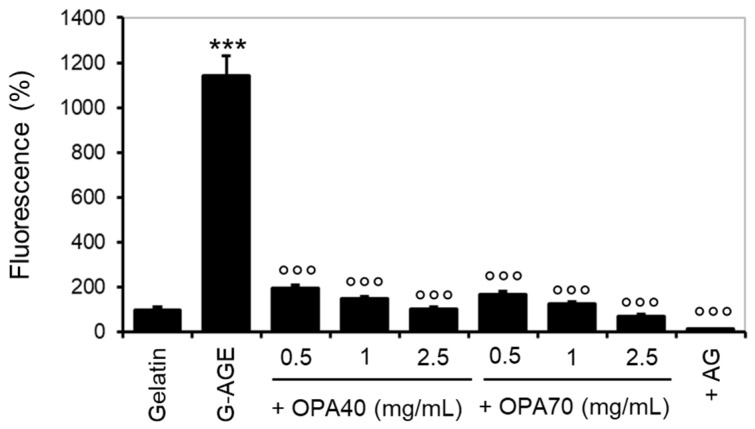
In vitro glycation of gelatin and the inhibitory effect of the OPA40 and OPA70 on G-AGE formation. Relative fluorescence intensity (λ_ex_/λ_em_ 335/385 nm) of unglycated gelatin, G-AGE, and G-AGE with OPA40 (0.5–2.5 mg/mL), OPA70 (0.5–2.5 mg/mL), or AG (10 mM) obtained at 45 °C for 72 h. All values are reported as percentage ratio with respect to unglycated gelatin. Data are the mean of three independent experiments. Error bars represent standard deviation. Tukey’s test: *** *p* < 0.001 vs. unglycated gelatin; °°° *p* < 0.001 vs. G-AGE formed in the absence of the extracts.

As shown in Figure 4, after 72 h of incubation between gelatin and Glc, there was an approximately 11-fold (1142 ± 88) peak fluorescence intensity of G-AGE compared to the intensity of unglycated gelatin. Both OPA extracts inhibited G-AGE formation in a dose-dependent manner, as represented in Figure 4. Specifically, OPA40 inhibited G-AGE formation by approximately 6-fold (193 ± 15%), 8-fold (147 ± 13%), and 11-fold (103 ± 8%) at the concentration of 0.5, 1, and 2.5 mg/mL, respectively (corresponding to 0.2 mg/mL, 0.4 mg/mL, and 1 mg/mL of Ole, respectively). Comparably, OPA70 reduced G-AGE formation by 7-fold (169 ± 13%), 9-fold (126 ± 10%), and 15-fold (71 ± 15%) at the concentration of 0.5, 1 mg/mL, and 2.5 mg/mL, respectively (corresponding to 0.35 mg/mL, 0.7 mg/mL, and 1.75 mg/mL of Ole). However, the anti-glycation effect of the two OPA-EXTs was lower than that of AG (10 mM), used as a control, which strongly reduced G-AGE formation by about 85-fold (13 ± 1%).

As gelatin is a heterogeneous mixture of collagen proteins with an average high molecular weight, the fluorescence data obtained could not be confirmed by N-PAGE electrophoretic migration for G-AGE.

Over time, hyperglycosylated collagens accumulate in the basement membrane and in the skin, causing morphological changes. As a consequence, AGE accumulation alters the structure of collagen fibrils and impairs collagen metabolism, thereby reducing bone turnover and preventing skeletal repair and adaptation. Among the known serious consequences mediated by hyperglycosylated collagen are diabetic foot and problematic wound healing processes [38]. Consequently, identifying compounds that can limit collagen hyperglycosylation offers the possibility of modifying collagen-mediated consequences in diabetes. 

In this regard, the results obtained in this work suggest the potential use of both OPA-EXTs as protein anti-glycation natural agents.

#### 2.3.3. The Inhibitory Effect of TTP70 Compared with OA in the In Vitro Albumin Glycation System (A-AGE)

In order to evaluate the effect of *Olea europaea* L. leaf extracts on the protein glycation process, an olive leaf extract enriched in pentacyclic triterpenes (TTP70) was also used in this work. The TTP70 contained 65.34 ± 1.06% *w*/*w* TTPs, which were found to correspond mainly to OA. 

Therefore, the anti-glycation effect of TTP70 was compared to the anti-glycation effect of the single pentacyclic triterpenoid OA in the in vitro albumin glycation system (A-AGE).

To assess the effect of TTP70 on albumin glycation, A-AGE formation was examined by incubating BSA (1 mg/mL) and Glc (500 mM) at 60 °C for 72 h in the presence of 0.3 and 0.4 mg/mL of TTP70. The formation of A-AGE in the presence of the extract was compared to the formation of A-AGE in the presence of the major constituent of the extract, i.e., OA at concentrations of 0.15 and 0.2 mg/mL. Note that TTP70 and OA showed no autofluorescence at the wavelengths used for A-AGE detection.

**Figure 5:**
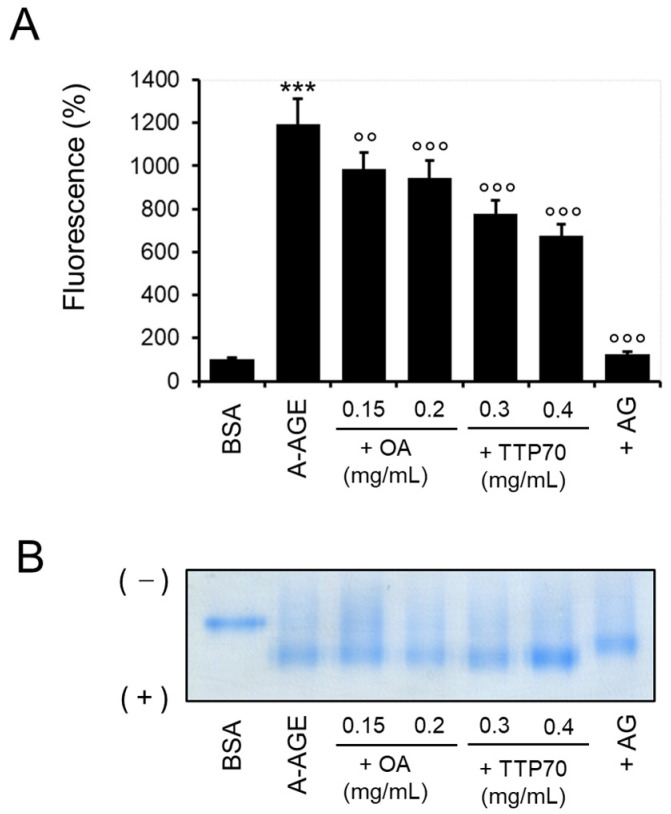
In vitro glycation of albumin and the inhibitory effect of TTP70 and OA on A-AGE formation. (**A**) Relative fluorescence intensity (λ_ex_/λ_em_ 335/385 nm) of unglycated albumin (BSA), A-AGE, and A-AGE obtained in the presence of OA (0.15 and 0.2 mg/mL), TTP70 (0.3 and 0.4 mg/mL), or AG (10 mM) at 60 °C for 72 h. All values are reported as percentage ratio to unglycated albumin (BSA). Data are the mean of three independent experiments. Error bars represent standard deviation. Tukey’s test: *** *p* < 0.001 vs. unglycated BSA; °° *p* < 0.01, °°° *p* < 0.001 vs. A-AGE formed in the absence of OA or TTP70. (**B**) Representative N-PAGE image of unglycated BSA and A-AGE obtained at 60 °C for 72 h in the presence or absence of OA and TTP70 extracts. A-AGE obtained in the presence of AG was used as a control for protein glycation inhibition. The symbols (+) and (−) represent the anode and cathode in the N-PAGE gel, respectively.

As shown in Figure 5A, after 72 h of incubation between BSA and Glc, there was an approximately 11-fold (1193 ± 117) peak fluorescence intensity of A-AGE compared to the intensity of unglycated albumin (BSA). In the presence of TTP70, A-AGE formation was reduced in a concentration-dependent manner by 1.5-fold (778 ± 60%) and 1.7-fold (677 ± 52%) with 0.3 and 0.4 mg/mL extract, respectively. Concentrations of 0.15 and 0.2 mg/mL OA, the major constituent of TTP70, caused a smaller reduction in A-AGE formation than TTP70. In particular, OA caused an approximately 1.2-fold reduction (987 ± 77% and 943 ± 80%, at concentrations of 0.15 and 0.2 mg/mL, respectively) compared to A-AGE. Note that the fluorescence emission of the obtained glycation products was not interfered by the resuspension medium of the extract, i.e., ethanol, at the used concentrations. Considering these results, the anti-glycation effect of TTP70 could be attributable not only to the major constituent OA, but to the synergistic action of other triterpenes and other constituents of the extract. Although the inhibitory effect of TTP70 and OA on the glycation of albumin is visible by detecting the autofluorescence intensity of the glycation products, the percentage of inhibition remains rather low to be appreciated by the N-PAGE technique. In fact, as shown in Figure 5B, the difference in migration between A-AGE and the glycation products obtained in the presence of TTP70 and OA is hardly perceptible. 

Serum albumin binds and transports many endogenous and exogenous substances, such as hormones, fatty acids, drugs, and other molecules. The literature reports that TTPs can firmly bind to human serum proteins (mainly albumin) via hydrogen bonds, van der Waals forces, and hydrophobic interactions [39]. This shows that the hydrophobic TTP70 extract, enriched in triterpenes, can tightly bind to albumin and act as an inhibitor of protein glycation in vitro, although to a lesser degree than OPA-EXT described above. The strong interaction with albumin makes TTP70 a promising anti-glycation agent in vivo as well. These results align with the literature on the anti-glycating role of TTPs, particularly OA. However, this effect has been reported in an in vitro fructose glycation system [40]. Therefore, in this work, the anti-glycation effect of OA in an in vitro glucose glycation system was evaluated for the first time. These results are significant because glucose is the main circulating sugar in the blood and thus the primary biological source of protein glycation, especially under conditions of hyperglycemia or diabetes.

#### 2.3.4. The Inhibitory Effect of TTP70 Compared with OA in the In Vitro Gelatin Glycation System (G-AGE)

**Figure 6:**
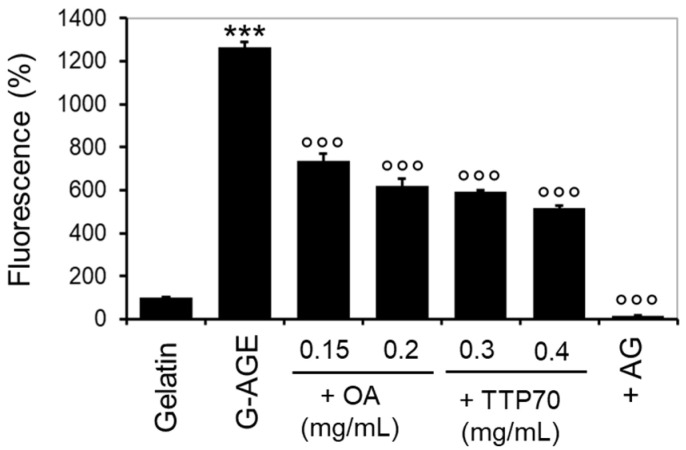
In vitro glycation of gelatin and the inhibitory effect of the TTP70 and OA on G-AGE formation. Relative fluorescence intensity (λ_ex_/λ_em_ 335/385 nm) of unglycated gelatin, G-AGE, and G-AGE with OA (0.15–0.2 mg/mL), TTP70 (0.3–0.4 mg/mL), or AG (10 mM) obtained at 45 °C for 72 h. All values are reported as percentage ratio with respect to unglycated gelatin. Data are the mean of three independent experiments. Error bars represent standard deviation. Tukey’s test: *** *p* < 0.001 vs. unglycated gelatin; °°° *p* < 0.001 vs. G-AGE formed in the absence of the extracts.

The effect of TTP70 on protein glycation was also evaluated on the gelatin glycation system in vitro (G-AGE). Specifically, the formation of G-AGE was examined by incubating at 45 °C for 72 h gelatin (1 mg/mL) and Glc (500 mM) in the presence of 0.3 and 0.4 mg/mL of TTP70. The formation of A-AGE in the presence of the extract was compared to the formation of G-AGE in the presence of OA at concentrations of 0.15 and 0.2 mg/mL. As shown in Figure 6, after 72 h of incubation between gelatin and Glc, there was an approximately 12-fold (12,662 ± 23) peak in the fluorescence intensity of G-AGE compared to the intensity of gelatin. 

TTP70 inhibited the formation of G-AGE approximately 2-fold (590 ± 10%) and 2.5-fold (517 ± 9%) at the concentration of 0.3 and 0.4 mg/mL, respectively. The effect of OA at the concentrations of 0.15 and 0.2 mg/mL on gelatin glycation was roughly comparable to that of the extract. In fact, OA reduced G-AGE formation by approximately 1.7-fold (737 ± 33%) and 2-fold (618 ± 33%). The anti-glycation effect of TTP70 and OA was less than that of AG (10 mM), used as the control, which strongly reduced G-AGE formation by about 86-fold (14 ± 1%).

To the best of our knowledge, for the first time, the in vitro anti-glycation effect of an olive leaf extract, enriched with TTPs, and in particular OA, has been tested on an in vitro glycation system in the presence of glucose and gelatin. As already discussed, the importance of an anti-glycation effect of natural products on collagen, as the main structural protein in the ECM, stems from the key role this protein substrate plays in the formation of glycation products under hyperglycemic or diabetic conditions, and the important consequences that can result from a hyperglycosylated, and thus functionally impaired, collagen.

## 3. Materials and Methods 

### 3.1. Chemicals and Reagents

Bovine serum albumin (BSA), gelatin from porcine skin (Type A), D-glucose (Glc), aminoguanidine (AG), and all other chemicals were purchased from Sigma Aldrich-Merck. Electrophoresis reagents and Coomassie Brilliant Blue G were provided by Bio-Rad (Hercules, CA, USA). Disposable plastics were from Sarstedt (Nümbrecht, Germany). 

Oleanolic acid (OA, >97%) and *Olea europaea* L. leaf extracts (EXTs) were supplied by Natac Biotech SL (Getafe, Madrid, Spain). Oleanolic acid, maslinic acid, ursolic acid, erythrodiol, uvaol, luteolin-7-O-glucoside, and apigenin-7-O-glucoside were purchased from Extrasynthese (Genay, Cedex, France). Oleuropein, hydroxytyrosol, verbascoside, luteolin were from Sigma-Aldrich, Darmstadt, Germany. All analytical-grade and HPLC-grade solvents were provided by Sigma Aldrich Italia (Milan, Italy). Distilled water was obtained from a Simplicity UV Water Purification System from Merck Millipore (Darmstadt, Germany).

### 3.2. Preparation of Olea europaea L. Extracts (EXTs)

#### 3.2.1. TTP70 Preparation

Leaves from *Olea europaea* L. were harvested in October of 2021. The leaves were collected from an olive oil farm located near Palenciana (Cordoba, Spain), variety Picual. After collection, the olive leaves were air-dried until the required moisture content was reached. The initial moisture content of the olive leaves was 42.57%. Before extraction, the leaves were ground to small pieces with 250–500 µm particle size. The extraction was performed with methanol, plant solvent ratio 1:50. Then, the extract was concentrated, and the triterpenes were precipitated out of solution by adding H_2_O. The precipitate was dried, and then washed with a non-polar solvent. The solid fraction contained approximately 70% of triterpenes. The triterpene-rich solid was brought to dryness by oven drying at 80 °C and homogenized by milling. The final product was obtained as a fine white powder. 

#### 3.2.2. OPA40 and OPA70 Preparation

Leaves from *Olea europaea* L. were harvested in May of 2022. The leaves were collected from an olive oil farm, near Benacazón (Sevilla, Spain), variety Manzanilla sevillana. After picking, the olive leaves were sun-dried until the required moisture content was reached. The initial moisture content of the olive leaves was 47.72%. Before extraction, the leaves were ground to small pieces with 250–500 µm particle size. The extraction was performed with a hydroalcoholic solution, plant solvent ratio 1:20. Then, the extract was concentrated and fractionated by column chromatography using an industrial macroporous adsorption resin. The fraction enriched in oleuropein was brought to dryness under reduced pressure at 40 °C and homogenized by milling to obtain the OPA40 extract. To obtain OPA70, the liquid fraction enriched in oleuropein was filtered by tangential ultrafiltration and the filtrate was brought to dryness under reduced pressure at 40 °C and homogenized by milling.

The botanical identification, which included a macroscopic and microscopic examination, as well as thin-layer chromatography analysis, was carried out by Dr. José Carlos Quintela in accordance with the Olive Leaf monograph (01/2022:1878) from the European Pharmacopoeia. An internal specimen is kept in the sample library of Natac Biotech, registry O4V-NAT-043 and 202110281236.

### 3.3. HPLC-DAD Analysis

A 1200 High-Performance Liquid Chromatograph equipped with a diode array detector (Agilent Technologies Italia Spa, Rome, Italy) was used for chromatographic determination. The analytical column was a Luna Omega Polar C18 (150 × 4.6 mm, 3 μm, Agilent Technology, Santa Clara, CA, USA). The analytical conditions for TTP determination have been previously reported [41,42]. The detection wavelength was 210 nm. The attribution and quantification of the peaks was obtained using the standard compounds.

The final extracts (OPA40 and OPA70) were characterized by HPLC-DAD and HPLC-MS/MS analyses, as previously reported [43,44,45,46,47]. Acquisition of the phenols was performed at 233 nm, 280 nm, 330 nm, and 355 nm. Oleuropein, hydroxytyrosol, verbascoside, luteolin, luteolin-7-O-glucoside, and apegienin-7-O-glucoside were used as internal standards.

### 3.4. In Vitro AGE Formation by Albumin–Glucose System (A-AGE)

**Scheme 1:**
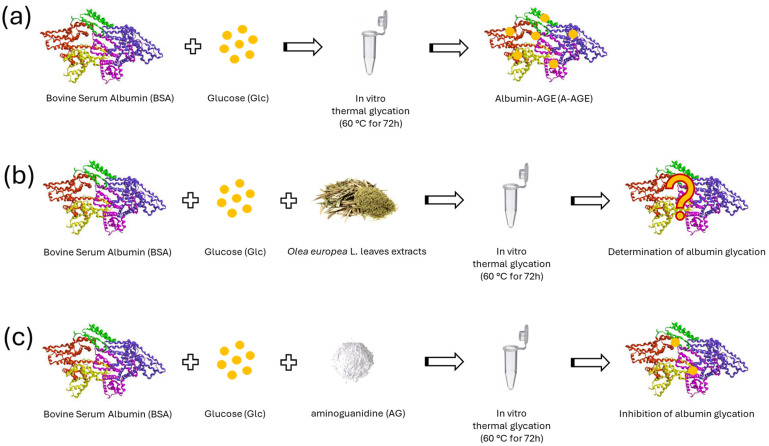
(**a**) In vitro AGE formation by albumin–glucose system (A-AGE), in the presence of (**b**) *Olea europea* L. leaf extracts or (**c**) aminoguanidine (AG). The image of the BSA was taken from the following website: https://www.chemicalbook.com/Article/Classification-of-bovine-serum-albumin.htm, accessed on 17 July 2024.

The AGEs were obtained in vitro by incubating BSA (1 mg/mL) with Glc (500 mM) in phosphate-buffered saline (PBS: pH 7.4, 1.37 M NaCl, 27 mM KCl, 100 mM Na_2_HPO_4_, 18 mM KH_2_PO_4_) at 60 °C for 72 h, under agitation (300 rpm), using a Thermo-Shaker TS-100 (Biosan, Riga, LV), and referred to as A-AGE below (Scheme 1a). The effect of *Olea europea* L. extracts on albumin glycation was assessed by incubating BSA with Glc in the presence of OPA40, OPA70, TTP70, and OA for 72 h at 60 °C (Scheme 1b). 

Positive control of the inhibition of A-AGE formation was performed by incubating BSA and Glc in the presence of aminoguanidine (AG; 10 mM), a known synthetic anti-glycation agent (Scheme 1c).

A-AGE formation was verified by measuring the autofluorescence of AGEs at λ_ex_/λ_em_ 335/385 nm [48] using a Biotek Synergy 1H plate reader.

The percentage of intrinsic fluorescence intensity of A-AGE was calculated as follows:

$$\;A\;-AGE(\%)=\frac{\left(BSA+Glc+{inhibitor\;}^{1})-(BSA+{inhibitor\;}^{1}\right)}{\left(BSA+Glc\right)-BSA}\times 100$$

^1^ Inhibitor: OPA40, OPA70, TTP70, OA or AG.

### 3.5. In Vitro AGE Formation by Gelatin–Glucose System (G-AGE)

**Scheme 2:**
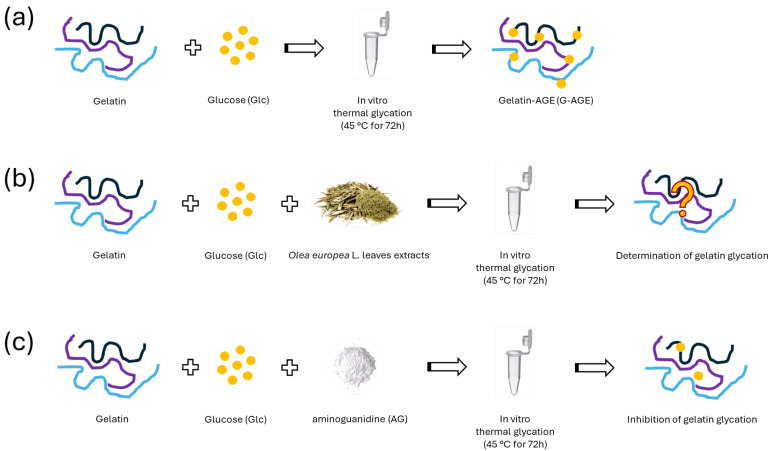
(**a**) In vitro AGE formation by gelatin–glucose system (G-AGE), in the presence of (**b**) *Olea europea* L. leaf extracts or (**c**) aminoguanidine (AG).

The AGEs were obtained in vitro by incubating gelatin (1 mg/mL) with Glc (500 mM) in phosphate-buffered saline (PBS: pH 7.4, 1.37 M NaCl, 27 mM KCl, 100 mM Na_2_HPO_4_, 18 mM KH_2_PO_4_) at 45 °C for 72 h, under agitation (300 rpm), using a Thermo-Shaker TS-100 (Biosan, Riga, LV), and referred to as G-AGE below (Scheme 2a). Gelatin solution without Glc was prepared as a control and incubated under the same conditions. 

The effect of *Olea europea* L. extracts on gelatin glycation was assessed by incubating gelatin with Glc in the presence of OPA40, OPA70, TTP70, and OA for 72 h at 45 °C (Scheme 2b). 

OPA40 and OPA70 extract were used at concentrations between 0.5 and 2.5 mg/mL, corresponding to 0.2–1 mg/mL and 0.35–1.75 mg/mL of Ole, respectively.

TTP70 extract was used at concentrations of 0.1 and 0.2 mg/mL, corresponding to 50–100 µg/mL of OA, and the single triterpenoid OA was used at concentrations of 50–100 µg/mL.

Positive control of the inhibition of G-AGE formation was performed by incubating gelatin and Glc in the presence of aminoguanidine (AG; 10 mM), as shown in Scheme 2c.

G-AGE formation was verified by measuring the autofluorescence of AGEs at λ_ex_/λ_em_ 335/385 nm [37] using a Biotek Synergy 1H plate reader.

The percentage of intrinsic fluorescence intensity of G-AGE was calculated as follows:

(1) $$G\;-AGE(\%)=\frac{\left(gelatin+Glc+{inhibitor\;}^{1})-(gelatin+{inhibitor\;}^{1}\right)}{\left(gelatin+Glc\right)-gelatin}\times 100$$

^1^ Inhibitor: OPA40, OPA70, TTP70, or AG.

### 3.6. Native Polyacrylamide Gel Electrophoresis (N-PAGE) 

The formation of A-AGE was detected by 10% N-PAGE under native conditions [Tris-glycine buffer: 25 mM Tris and 192 mM Glycine, pH 8.3]. 

In a dilution of 1:1, the protein samples were mixed with sample buffer [62.5 mM Tris-HCl pH 6.8, 25% (*w*/*v*) glycerol and 0.5% bromophenol blue]. No reducing agents were used in N-PAGE.

A-AGE samples (1 μg of proteins) were separated on N-PAGE for 80 min at constant 200 V. By following the manufacturer’s instructions, colloidal Coomassie Brilliant Blue G dye was used to stain the protein bands. 

## 4. Conclusions

The identification of natural products with anti-glycation properties supports the use of an integrated therapeutic approach aimed at improving the clinical condition of the diabetic patient, reducing the risk of occurrence of secondary complications related to the formation of glycation products and thus increasing life expectancies. In fact, diabetes is a complex clinical condition that requires a variety of interventions that go beyond simply controlling glycemia levels. The evaluation and treatment of chronic complications associated with diabetes are therefore equally important, including those associated with the formation of glycation products.

Since ancient times, medicinal plants have been used as a source of effective and valuable products for the development of alternative therapies. With the expansion of the concept of green chemistry and sustainable development, the use of environmentally friendly alternatives is gaining attention in the field of human health. Plant extracts are one such alternative. Recycling the co-products of processing these plant materials allows valuable bioactive compounds to be obtained from under-exploited materials, contributing to the revalorization of these wastes. Furthermore, plant leaf extracts have generally shown reasonably better efficacy at relatively low concentrations, and in addition, the preparation of a crude extract has a sustainable added value as it requires less use of solvents and chemical reagents than the purification of a single compound, even if natural. 

In conclusion, our study demonstrates that the olive leaf extracts OPA40 and OPA70 exhibited strong, dose-dependent anti-glycation effects, outperforming the synthetic inhibitor aminoguanidine. These effects were corroborated by electrophoresis and further supported by similar results in a gelatin–glucose system, establishing OPA40 and OPA70 as potent natural anti-glycation agents. Additionally, TTP70, the extract enriched in pentacyclic triterpenes, showed moderate anti-glycation activity, with a synergistic effect of its components. However, its efficacy was lower than that of OPA-EXTs. These findings suggest the potential of OPA40 and OPA70 in managing diabetes-related complications, while also highlighting the importance of further research on natural products for glycation inhibition.

This work emphasizes the significance of the anti-glycation activity of olive leaf extracts for human health, supporting a sustainable economy and environmental respect.

## Figures and Tables

**Table 1:** Quali-quantitative composition of OPA40 and OPA70 (%*w*/*w*).

Compound	OPA40	OPA70
Oleuropein	41.67	66.59
Hydroxytyrosol	0.16	0.31
Verbascoside	1.18	1.13
Oleuroside	4.39	5.85
Luteolin	0.02	0.04
Luteolin-7-O-glucoside	1.05	0.69
Luteolin-4-O-glucoside	0.46	0.44
Luteolin diglucoside	0.06	0
Apigenin-7-O-glucoside	0.17	0.27
Apigenin-7-O-rutinoside	0.13	0
7-epilogalin	0.30	0
Elenolic acid glucoside	0.04	0
Total	49.63	75.32

**Table 2:** Quali-quantitative composition of TTP70 (%*w*/*w*).

Compound	%*w*/*w*
Oleanolic acid	36.73
Maslinic acid	14.41
Uesolic acid	8.63
Erythrodiol	0.97
Uvaol	4.61
Total	65.34

## Data Availability

Data are contained within the article and Supplementary Materials.

## Supplementary Materials

The following supporting information can be downloaded at: https://www.mdpi.com/article/10.3390/molecules29184368/s1, Figure S1: chromatographic profiles of OPA40 extract at 233, 280, 330, and 355 nm and m/z of identified compounds; Figure S2: chromatographic profile of the TTP70 at 210 nm. Maslinic acid: 4.59 min, oleanolic acid: 8.56 min, ursolic acid: 8.72 min, uvaol: 12.16 min, erythrodiol 12.55 min.
