# Supplementary material for: Olea europaea L. Leaves as a Source of Anti-Glycation Compounds
Source: Molecules. 2024 Sep 14;29(18):4368. doi: 10.3390/molecules29184368 (PMC11434099; doi:10.3390/molecules29184368)
Supplement: Supplementary file 1 [file molecules-29-04368-s001.zip › molecules-3143773-supplementary.pdf]

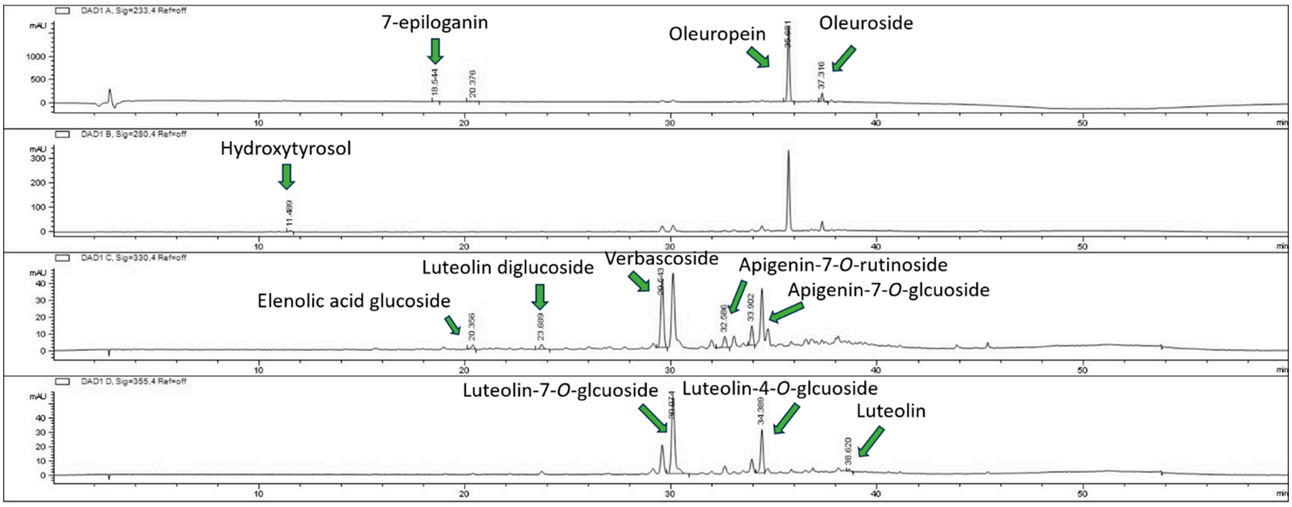

Rt (min)	*m/z* *[M-H]-*	Compound
11,48	*153.05*	Hydroxytyrosol
20,13	389,3	7-epiloganin
21,80	403,2	elenolic acid glucoside
25,25	609,2	Luteolin diglucoside
31,35	623,3	Verbascoside
31,83	447,1	Luteolin-7-*O*-glucoside
34,39	577,4	Apigenin-7-*O*-rutinoside
35,32	431,1	Apigenin-7-*O*-glucoside
35,7	447,1	Luteolin 4-O-glucoside
36,71	539,4	Oleuropein
38,27	539,4	Oleuroside
38,62	287,2	Luteolin

Figure S1. Chromatographic profiles of OPA40 extract at 233, 280, 330 and 355 nm and m/z of identified compounds.

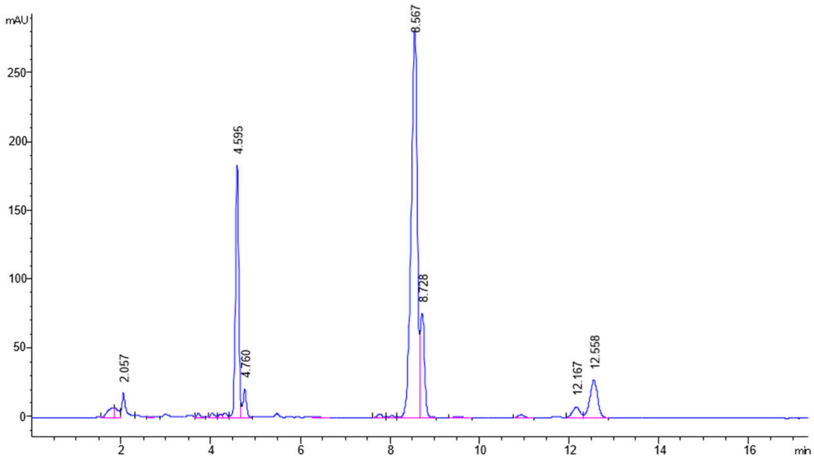

Figure S2. Chromatographic profile of the TTP70 at 210 nm. Maslinic acid: 4.59 min, oleanolic acid: 8.56 min, ursolic acid: 8.72 min, uvaol: 12.16 min, erythrodiol 12.55 min.
